# The Time to Discharge and Pain Reliever Intake Depending on the Use of a Drain After Laparoscopic Cholecystectomy

**DOI:** 10.3390/jcm14248752

**Published:** 2025-12-10

**Authors:** Łukasz Strzępek, Aleksandra Czerw, Mateusz Curyło, Marcin Mikos, Maciej Florek, Dorota Charkiewicz, Olga Partyka, Monika Pajewska, Grzegorz Stachacz, Katarzyna Sygit, Sławomir Porada, Izabela Gąska, Elżbieta Kaczmar, Elżbieta Grochans, Anna M. Cybulska, Daria Schneider-Matyka, Ewa Bandurska, Weronika Ciećko, Jarosław Drobnik, Piotr Pobrotyn, Dorota Waśko-Czopnik, Tomasz Sowiński, Julia Pobrotyn, Adam Wiatkowski, Tomasz Czapla, Monika Borzuchowska, Remigiusz Kozlowski

**Affiliations:** 1 Clinical Department of General and Oncological Surgery, Saint Raphael Hospital, 30-693 Cracow, Poland; 2 Department of Surgery, Andrzej Frycz Modrzewski Krakow University, 30-705 Cracow, Poland; 3 Department of Health Economics and Insurance, Medical University of Warsaw, 00-581 Warsaw, Poland; 4 Department of Economic and System Analyses, National Institute of Public Health NIH-National Research Institute, 00-791 Warsaw, Poland; 5 Institute of Health Sciences, University of the National Education Commission, 30-084 Krakow, Poland; 6 Medical Rehabilitation Department, The Ministry of the Interior and Administration Hospital, 30-053 Krakow, Poland; 7 Department of Bioinformatics and Public Health, Andrzej Frycz Modrzewski Krakow University, 30-705 Krakow, Poland; 8 Faculty of Medicine and Health Sciences, University of Kalisz, 62-800 Kalisz, Poland; 9 Faculty of Health Sciences and Psychology, Collegium Medicum, University of Rzeszów, 35-310 Rzeszow, Poland; 10 Medical Institute, Jan Grodek State University in Sanok, 38-500 Sanok, Poland; 11 Department of Nursing, Faculty of Health Sciences, Pomeranian Medical University in Szczecin, 71-210 Szczecin, Polandanna.cybulska@pum.edu.pl (A.M.C.);; 12 Center for Competence Development, Integrated Care and e-Health, Medical University of Gdansk, 80-204 Gdansk, Poland; 13 Department of Family Medicine, Faculty of Medicine, Wroclaw Medical University, 51-141 Wroclaw, Poland; 14 Citodent Dental Center, Furtak-Pobrotyn & Company Limited Partnership, 05-220 Olawa, Poland; 15 Department of Gastroenterology, Hepatology with Inflammatory Bowel Disease Subunit, Provincial Specialist Hospital J. Gromkowskiego, 51-149 Wroclaw, Poland; 16 Department of Non-Surgical Clinical Sciences Faculty of Medicine, Wroclaw University of Science and Technology, 50-370 Wroclaw, Poland; 17 Endocare Medical Center, Simple Joint-Stock Company (S.J.S.C.), 50-558 Wroclaw, Poland; 18 Faculty of Medicine, Wroclaw Medical University, 50-345 Wroclaw, Polandadam.wiatkowski@student.umw.edu.pl (A.W.); 19 Department of Management, Faculty of Management, University of Lodz, 90-237 Lodz, Poland; 20 Department of Management and Logistics in Healthcare, Medical University of Lodz, 90-131 Lodz, Poland; monika.borzuchowska@umed.lodz.pl (M.B.); remigiusz.kozlowski@umed.lodz.pl (R.K.)

**Keywords:** laparoscopic cholecystectomy, pain, hospital stay, pain reliever, drain

## Abstract

**Background:** Laparoscopic cholecystectomy is the standard surgical treatment for symptomatic gallbladder diseases. In clinical practice, it can be followed by drainage or not. The results acquired so far regarding drain insertion are mixed. The current study used data from medical records and aimed to provide results verifying whether there are differences between patients with postoperative drains inserted after laparoscopic cholecystectomy and the group of patients without postoperative drains, with regard to the time from surgery to discharge and the intake of pain relievers, while controlling for patients’ age and gender. **Methods:** Medical records regarding 209 patients, 151 females, and 58 males aged 20–83 were included in the analysis. **Results:** The time from surgery to discharge was significantly longer in the group of patients with a postoperative drain inserted, both in terms of days (1.27 vs. 1.05) and in terms of hours (29.48 vs. 23.04). Also, the amount of Pyralgin and Paracetamol used was significantly higher in the group of patients with a postoperative drain inserted (3.33 g vs. 2.41 g, and 3.40 g vs. 2.45 g, respectively). **Conclusions:** The results acquired are consistent with many other studies. However, some studies do not show these differences. Therefore, to provide a definitive answer, a meta-analysis followed by meta-regression is needed.

## 1. Introduction

Laparoscopic cholecystectomy is the standard surgical treatment for symptomatic gallbladder diseases. Since the 1990s, it has gradually replaced open cholecystectomy on the basis of its less invasive character. It is usually associated with lower postoperative pain, a shorter hospital stay, faster recovery, and fewer complications [1]. It is a method used for treating acute or chronic cholecystitis, symptomatic cholelithiasis, biliary dyskinesia, acalculous cholecystitis, gallstone pancreatitis, and gallbladder polyps. Contradictions for the surgery are the inability to tolerate general anesthesia, pneumoperitoneum, coagulopathy, and metastatic disease, because of the risk of exacerbating malignancy through procedures that may disrupt tumor integrity. However, laparoscopic cholecystectomy can also be used to treat gallbladder cancer in selected cases [2].

A drain may or may not be placed during a laparoscopic cholecystectomy. There is still an ongoing debate regarding the need for drainage. The results acquired so far are mixed. Some studies did not find any differences [3,4], some found drainage to be associated with adverse effects [5,6,7,8,9], and just one found some benefits [10]. Therefore, there is a need for further research, and the current study provides further results on the topic. A systematic collection of results will enable meta-analyses to be conducted in the future.

## 2. Materials and Methods

The current study is a retrospective study. The data were acquired from medical records. Therefore, no randomization was performed. The analysis aimed at providing answers to two research questions:

Are there differences between patients with a postoperative drain inserted after cholecystectomy and patients without a postoperative drain, with regard to the time from surgery to discharge, while controlling for patients’ age and gender?

Are there differences between patients with a postoperative drain inserted after cholecystectomy and patients without a postoperative drain, with regard to the intake of pain relievers, while controlling for patients’ age and gender?

Controlling for patients’ age and gender allowed for the use of more precise estimates.

### 2.1. Current Sample

The current sample consisted of the medical data records regarding 209 patients. The inclusion criteria were DC11 Cholelithiasis diagnosis and planned laparoscopic cholecystectomy performed between 03.01.2025 and 24.03.2025. Patients undergoing unplanned, urgent operations were excluded. The age of the patients ranged from 20 to 83 (M = 52.69; SD = 13.98), 151 females aged 24–83 (M = 51.26; SD = 13.38), and 58 males aged 20–80 (M = 56.40; SD = 14.93). Of these, 65 patients (31.1%), 45 females and 20 males aged 27–80 (M = 53.75; SD = 13.52), had a postoperative drain inserted after cholecystectomy, and 144 patients (68.9%), 106 females and 38 males aged 20–83 (M = 52.21; SD = 14.1621), did not have a postoperative drain inserted. According to the values of the independent samples *t*-test, the two groups did not differ significantly in terms of age, t(207) = −0.74, *p* > 0.05. According to the values of Pearson’s chi-squared test of independence, the two groups did not differ regarding patients’ gender as well, χ^2^(1) = 0.43, *p* > 0.05. In the case of 205 patients (98.1%), 140 patients without a surgery drain inserted (97.2%), and all patients with a surgery drain inserted, pain relievers were administered.

### 2.2. Data Analysis

The significance of relationships between the patient’s age and both the time from surgery to discharge and pain reliever intake was assessed with the use of Pearson’s correlation coefficients. The differences between female and male patients were verified with the use of Student’s *t*-test with Cohen’s d effect size measure.

In order to perform comparisons between the two groups of patients in terms of the time from surgery to discharge and pain reliever intake, we performed multivariate analysis of covariance (MANCOVA). This statistical method allowed for the comparison of the two groups while controlling for the age and gender of the patients included in the analysis. The time from surgery to discharge was measured in days and hours, and the intake of Pyralgin and Paracetamol was analyzed separately. We did not analyze the intake of Ibuprofen and Ketoprofen, because the majority of patients were not treated with these two pain relievers. Only 25 patients were treated with Ibuprofen, and just 5 with Ketoprofen (see Table 1). The results of MANCOVA were followed by an analysis of covariance (ANCOVA) to determine which variables differed significantly. Also, eta squared size effect measures were calculated.

## 3. Results

Table 1 depicts descriptive statistics for the doses of pain relievers used.

The time from surgery to discharge ranged from 2 to 6 days (M = 1.12; SD = 0.53) or from 11 to 140 h (M = 25.08; SD = 13.29). The drain was inserted in cases with perforation of the gallbladder during the procedure and leakage of bile or bleeding from the residual bed, as decided by the operator. The maximum time for the drain removal was 48 h after the operation. The time and reason for removal depended on the operator; the reasons included if the content is not bloody or bilious, if the patient is asymptomatic, feels well, and tolerates a full oral diet, and if the amount of drainage is less than 100 mL in the first 24 h after the procedure. No standard post-op pain protocol was developed at the hospital.

### 3.1. Postoperative Drain Insertion, and the Time from Surgery to Discharge

Patients’ age correlated positively with the time from surgery to discharge measured in hours, r(207) = 0.155, *p* = 0.013. The correlation with the time from surgery to discharge, measured in days, was statistically insignificant, r(207) = 0.112, *p* = 0.053. The difference between male and female patients in terms of the time from surgery to discharge was statistically significant, both when it was measured in days, t(207) = −2.39, *p* = 0.009, d = −0.37, and when it was measured in hours, t(207) = −2.20, *p* = 0.014, d = −0.34. The average time from surgery to discharge for female patients was equal to 1.07 days (SD = 0.32) or 23.8 h (SD = 8.29), and it was shorter than the average time from surgery to discharge for male patients, which was equal to 1.26 days (SD = 0.85) or 28.28 h (SD = 20.91). We controlled for the differences involving both patients’ age and gender in the subsequent analysis.

According to the results of multivariate analysis of covariance, the differences between the group of patients with a postoperative drain inserted after cholecystectomy and the group of patients without a postoperative drain were statistically significant, F(2204) = 7.27, *p* = 0.001, η^2^ = 0.067. Table 2 depicts the estimated marginal mean values of the time from surgery to discharge, both in days and in hours, in the group of patients with a postoperative drain inserted and in the group of patients without a postoperative drain, with the values of the ANCOVA and effect size measures.

**Figure 1:**
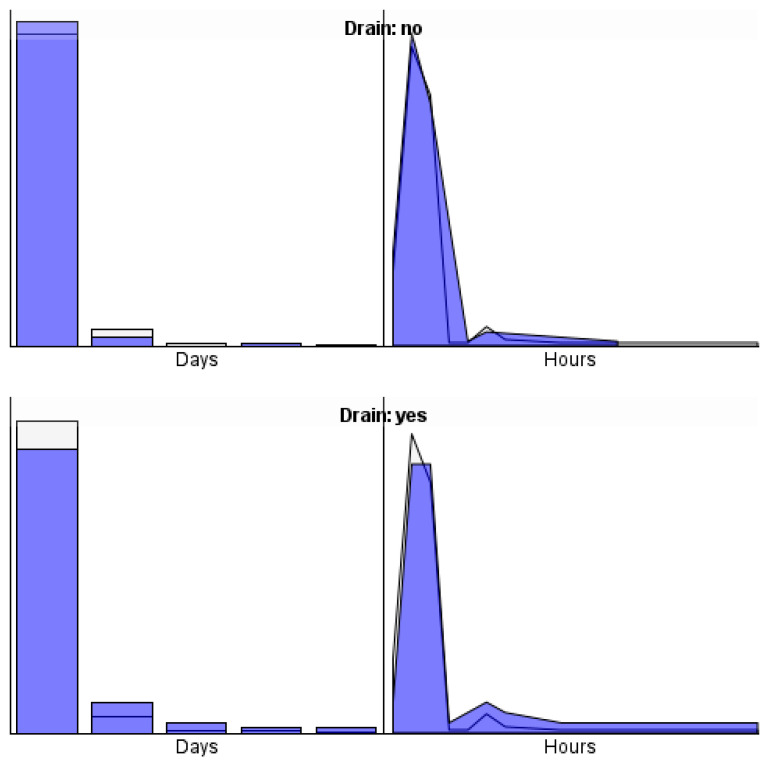
The time from surgery to discharge, in terms of days and hours in the group of patients with a postoperative drain inserted and in the group of patients without a postoperative drain.

The answer to the first research question of the current study is that the time from surgery to discharge was significantly longer in the group of patients with a postoperative drain inserted, both in terms of days and in terms of hours (see Figure 1).

### 3.2. Postoperative Drain Insertion and the Intake of Pain Relievers

Patients’ age correlated positively with the intake of Pyralgin, r(198) = 0.123, *p* = 0.013, and with the intake of Paracetamol, r(198) = 0.130, *p* = 0.034. The difference between male and female patients in terms of Pyralgin intake was statistically significant, t(196) = −1.90, *p* = 0.029, d = −0.30. The average intake in grams was higher in the group of male patients (M = 3.13; SD = 3.33) than in the group of female patients (M = 2.49; SD = 1.47). The difference between male and female patients in terms of Paracetamol intake was statistically insignificant, t(196) = −1.29, *p* = 0.099, d = −0.20. We controlled for the differences involving both patients’ age and gender in the subsequent analysis regarding pain reliever intake as well.

According to the results of multivariate analysis of covariance, the differences between the group of patients with a postoperative drain inserted after cholecystectomy, and the group of patients without a postoperative drain were statistically significant, F(2187) = 4.41, *p* = 0.013, η^2^ = 0.045. Table 3 depicts the estimated marginal mean values of grams of Pyralgin and Paracetamol used in the group of patients with postoperative drain inserted, and in the group of patients without postoperative drain, with the values of ANCOVA, and effect size measures. We did not include the intake of Ibuprofen and Ketoprofen, because only a small number of patients were treated with these two pain relievers.

The response to the second research question is that the amount of Pyralgin and Paracetamol used was significantly higher in the group of patients with a postoperative drain inserted.

## 4. Discussion

The results of the current study should be compared with the results of other similar studies regarding the benefits and adverse effects of drainage. In a retrospective study based on 457 cases of acutely inflamed gallbladder (226 with drains and 231 cases without drains), no statistically significant differences were found in terms of operating time, pain intensity measured with the use of visual analog scales, and the length of hospital stay [3]. Also, no effect on the postoperative morbidity was found. The authors conclude that there is no reason for inserting a drain routinely. In a sample of patients with acute calculous cholecystitis, no differences regarding the presence of subhepatic fluid collection at abdominal ultrasonography, postoperative abdominal and shoulder tip pain, use of analgesics, and morbidity were found depending on whether drainage was applied or not [4]. Also, the review in Cochrane Library shares the conclusion that currently there is no evidence to justify the routine use of drains after laparoscopic cholecystectomies [5]. On the basis of 12 trials, no differences in terms of short-term mortality, serious adverse events, the quality of life, the return to normal activity, and return to work were found. The proportion of patients who were discharged as a one-day procedure was significantly lower, and the operating time was significantly longer in the drain group. In a controlled randomized trial, with 284 patients randomized to have a drain placed and 281 patients randomized not to have a drain, no statistically significant differences in morbidity or the length of hospital stay were observed [6]. However, the drainage was associated with increased pain measured on a visual analog scale. In a prospective randomized study, involving 50 cases with drains and 50 cases without drains, no difference regarding the average operative time was found [10]. The number of cases with nausea and vomiting was higher in the no-drain group than in the drain group. Shoulder tip pain intensity was lower in the drain group in the first 12 h. However, after 12 h, the drain group had higher shoulder tip pain than the no-drain group. No statistically significant difference regarding analgesic requirement was detected. The length of hospital stay was longer in the drain group. In a study based on 60 cases, the drainage was found to be associated with significantly longer operative time, significantly higher operative pain, and significantly longer hospital stay [7]. A systematic review and meta-analysis found no differences in terms of morbidity, infection rate, and abdominal abscess rate [8]. On the other hand, the drainage was found to be associated with increased pain 24 h after the surgery, a longer hospital stay, and slower postoperative recovery. Moreover, late drain removal was found to be associated with a longer hospital stay and with a higher risk of surgical site infection [9]. Not only are drains not recommended even after complicated laparoscopic cholecystectomy for acute cholecystitis, but also if they are placed, they should be removed early.

The results of the current study showed that the time from surgery to discharge was significantly longer in the group of patients with postoperative drain inserted, which is consistent with some of the research cited above [4,7,8,10], but inconsistent with the other studies [3,6]. The other studies detected no differences depending on whether a drain was inserted or not, but no study acquired results indicating that the hospital stay was longer when a drain was not inserted. The results of the current study also showed that the amount of analgesics used was significantly higher in the group of patients with a drain inserted. This is consistent with research results cited above [6,7,8], and not consistent with other studies [3,5,10].

The two effects acquired in the current study, i.e., extended hospital stay and analgesic requirement, were also found in a study based on 130 patients undergoing elective laparoscopic cholecystectomy [11]. The difference regarding the length of hospital stay was even bigger. The mean duration of hospital stay was 4.06 days in the drain group and 2.26 days in the no-drain group. The authors also found a significantly longer duration of surgery, a higher incidence of postoperative nausea and vomiting, and a higher rate of patients who developed pain after surgery. In cases of uncomplicated laparoscopic cholecystectomy, the drainage was found to be associated with a longer hospital stay [12,13] and with a higher postoperative pain intensity measured with a visual analog scale [14]. Similarly, the pain intensity was lower in the group of patients without drainage after laparoscopic cholecystectomy in acute calculous cholecystitis [15]. The effects of drainage on the length of hospital stay and pain were also found in a study examining prophylactic drainage in cholecystectomy in uncomplicated acute cholecystitis [16]. Both were higher in the group of patients with prophylactic drains inserted. In addition, the effects were found in both cases where prophylactic drainage followed a laparoscopic cholecystectomy and an open cholecystectomy. Another study [17] examined the use of tube drains and corrugated drains. Postoperative pain was the least intense, and the hospital stay was the shortest in the group of patients without drains inserted. The authors concluded that drainage could be safely limited to specific cases assessed by the surgeon. In a prospective interventional study, the drain insertion was not randomized, but based on surgeon preference [18]. The most frequent reasons for drain placement were intraoperative hemorrhage and difficult operation. The length of hospital stay and the operation time were shorter, and the postoperative morbidity rate was lower in the group of patients without drains inserted. Also, no statistically significant difference in terms of postoperative complications was detected. In a randomized controlled trial evaluating morbidity after laparoscopic cholecystectomy for an acutely inflamed gall bladder and whether or not a drain was inserted, a longer hospital stay and a higher pain intensity evaluated with a visual analog scale were found in the group of patients with drainage [19].

The current study shows negative effects of drainage in terms of the time from surgery to discharge, and the use of analgesics, and is consistent with many other research results. However, every study based on a sample of patients has limitations. The heterogeneity of patients included in the analysis seems to be the main issue. The samples consisting of patients with different types of diagnosis, and with different courses of laparoscopic cholecystectomy, may have different consequences of a drain insertion. Also, in the current study, no matching regarding the pathological grading of the inflammation between the two groups compared was performed.

## 5. Conclusions

The results of the current study show adverse effects of drainage regarding the time from surgery to discharge and the use of analgesics. To obtain a definitive answer to the questions regarding the need for drainage and the consequences of inserting a drain in the context of laparoscopic cholecystectomy, a meta-analysis followed by meta-regression is needed. The type of diagnosis and comorbidities, as they are potential predictors of longer hospital stay [20], as well as the reasons for the drain placement and the time from the drain insertion to drain removal, should be considered as potential moderators of the drainage effect sign and strength.

## Figures and Tables

**Table 1:** Descriptive statistics for the doses of pain relievers used.

Drug	Unit	*M*	*SD*	*min*	*max*	*n*
Pyralgin	g	2.67	2.17	1	20	198
Paracetamol	g	2.73	2.12	1	20	198
Ibuprofen	mg	600.00	500.00	200	2800	25
Ketoprofen	mg	100.00	0.00	100	100	5

*M*—mean value; *SD*—standard deviation; *min*—minimum value; *max*—maximum value; *n*—number of patients treated.

**Table 2:** Estimated marginal mean values of the time from surgery to discharge in the group of patients with a postoperative drain inserted and in the group of patients without a postoperative drain.

	Postoperative Drain							
Time from	no		yes					
surgery to discharge	*M*	*SE*	*M*	*SE*	*F*	*df*	*p*	η^2^
Days	1.05	0.04	1.27	0.06	7.94	1205	0.005	0.037
Hours	23.04	1.05	29.48	1.57	11.56	1205	0.001	0.053

*M*—mean value; *SE*—standard error; *F*—analysis of covariance test; *df*—degrees of freedom; *p*—statistical significance; η^2^—eta squared size effect measure.

**Table 3:** Estimated marginal mean values of grams of Pyralgin and Paracetamol used in the group of patients with a postoperative drain inserted and in the group of patients without a postoperative drain.

	Postoperative Drain							
	no		yes					
Pain relievers	*M*	*SE*	*M*	*SE*	*F*	*df*	*p*	η^2^
Pyralgin (g.)	2.41	0.19	3.33	0.27	7.65	1.188	0.006	0.039
Paracetamol (g.)	2.45	0.18	3.40	0.27	8.80	1.188	0.003	0.045

*M*—mean value; *SE*—standard error; *F*—analysis of covariance test; *df*—degrees of freedom; *p*—statistical significance; η^2^—eta squared size effect measure.

## Data Availability

Data are available upon request from the authors.
